# Sudden Infant Death Syndrome and prenatal maternal smoking: rising attributed risk in the *Back to Sleep *era

**DOI:** 10.1186/1741-7015-3-4

**Published:** 2005-01-11

**Authors:** Mark E Anderson, Daniel C Johnson, Holly A Batal

**Affiliations:** 1 Department of Community Health Services, Division of Pediatrics, Denver Health and Hospitals Authority, Denver, Colorado, USA; 2 Division of General Internal Medicine, University of Colorado Health Sciences Center, Denver, Colorado, USA; 3 Department of Community Health Services, Division of General Internal Medicine, Denver Health and Hospitals Authority, Denver, Colorado, USA

**Keywords:** Sudden Infant Death Syndrome, SIDS, smoking, infant death, attributed risk

## Abstract

**Background:**

Parental smoking and prone sleep positioning are recognized causal features of Sudden Infant Death. This study quantifies the relationship between prenatal smoking and infant death over the time period of the *Back to Sleep *campaign in the United States, which encouraged parents to use a supine sleeping position for infants.

**Methods:**

This retrospective cohort study utilized the Colorado Birth Registry. All singleton, normal birth weight infants born from 1989 to 1998 were identified and linked to the Colorado Infant Death registry. Multivariable logistic regression was used to analyze the relationship between outcomes of interest and prenatal maternal cigarette use. Potential confounders analyzed included infant gender, gestational age, and birth year as well as maternal marital status, ethnicity, pregnancy interval, age, education, and alcohol use.

**Results:**

We analyzed 488,918 birth records after excluding 5835 records with missing smoking status. Smokers were more likely to be single, non-Hispanic, less educated, and to report alcohol use while pregnant (p < 0.001). The study included 598 SIDS cases of which 172 occurred in smoke-exposed infants. Smoke exposed infants were 1.9 times (95% CI 1.6 to 2.3) more likely to die of SIDS. The attributed risk associating smoking and SIDS increased during the study period from approximately 50% to 80%. During the entire study period 59% (101/172) of SIDS deaths in smoke-exposed infants were attributed to maternal smoking.

**Conclusions:**

Due to a decreased overall rate of SIDS likely due to changing infant sleep position, the attributed risk associating maternal smoking and SIDS has increased following the *Back to Sleep *campaign. Mothers should be informed of the 2-fold increased rate of SIDS associated with maternal cigarette consumption.

## Background

Previous literature has shown a relationship between maternal smoking and the Sudden Infant Death Syndrome (SIDS). Published studies vary in size and methodology but consistently demonstrate a two-fold increased odds of SIDS with both prenatal and postnatal maternal smoking [1-10], including one study of over 300,000 infants in an analysis of birth registry data from the late 1970s [4,6]. A recent secular change, namely the *Back to Sleep *campaign, has had a major role in reducing SIDS rates. This public health campaign encourages parents to place infants in a supine rather than prone sleeping position. Early studies investigating the effects of supine sleeping revealed significantly reduced SIDS rates [11], but smoking among mothers, which was not targeted as the primary intervention, remained unchanged.

The aim of this study was to confirm the relationship between reported prenatal maternal smoking and SIDS and to examine the effect of sleeping position changes on the attributed risk of SIDS and smoking. The analysis spans the rollout of the *Back to Sleep *campaign in the United States for the ten-year period from 1989 to 1998. We hypothesized that maternal prenatal smoking confers a clinically significant risk of SIDS and that an increased attributed risk of SIDS associated with smoking could be identified in the wake of the supine sleeping campaign.

## Methods

### Study setting/population

Since 1969, the State of Colorado has collected data on multiple items through a birth registry. This registry includes extensive demographic data such as maternal report of the number of cigarettes smoked per day during pregnancy. We used the Colorado Infant Death Registry over a 10-year period (1989–1998) to identify causes of infant death.

### Study design

We conducted a retrospective cohort study utilizing Cochran Mantel-Haenszel univariate analysis of risk factors and multiple logistic regression on the outcomes of infant death, SIDS, and respiratory deaths. We excluded non-singleton births (14978 records), infants born at less than 2500 grams (36779 records), and birth records where a mother's smoking status was unknown (5835 records). Additional records with missing data were coded so as to place the record in the presumed lower risk group: 42 records with unclear marital status were coded as "married," 67 records missing a maternal age were coded as age "18–34 years," 865 records with unclear gestational age were coded as "term" infants, and 4 records missing a gender assignment were coded as "female" in the analysis. Missing data for education (9659 records) and ethnicity (582 records) were coded as such and included in the analysis directly. We linked the birth and death registries using a unique birth number present in both registries. Utilizing multiple logistic regression, we modeled the exposure of interest, maternal smoking, as both a dichotomous and a continuous variable. Other variables analyzed included infant gender and gestational age (less than 37 weeks, 37 weeks or older), as well as maternal marital status, ethnicity, time between pregnancies (less than 12 months or 12 months and greater), maternal age (<18 years, 18 to 34 years, and >34 years), education, and self-reported use of alcohol in pregnancy. In the analyses for SIDS and respiratory causes of death, deaths from other causes were excluded from the analysis.

Power to detect a 20% difference in a baseline disease occurrence of 3 per 1000 was calculated at 99% for a population of 500,000. SAS version 8.0 (SAS Institute) on a PC was utilized for statistical analyses. We included the interaction between ethnicity and cigarette use in the modeling and requested the Hosmer-Lemeshow statistic from the model.

### Study outcomes

We compared cases of SIDS (ICD 9 codes 798.0 to 798.9) between cohorts of infants born to mothers who reported prenatal smoking versus mothers who reported no prenatal smoking. Secondary outcomes included total infant mortality and respiratory deaths (ICD 9 codes 033.0 to 033.9 and 460 to 490). We used unadjusted annual rates to calculate the attributable risk of SIDS associated with maternal cigarette consumption.

## Results

Over the 10-year study period, 1573 infants died, 598 SIDS cases occurred (1.2/1000 live born infants), and 34 infants died due to a respiratory etiology. Table 1 describes characteristics of the exposed and unexposed infant cohorts. Smoking mothers were statistically more likely to be single, non-Hispanic, less educated, and to report alcohol use in pregnancy.

**Table 1:** Demographic characteristics of 488,918 births according to maternal smoking habits during pregnancy, Colorado Birth Registry, 1989 to 1998.

	**Number (%)**	**Reported maternal smoking (%)**
**Sex**		
**male**	252,066 (51.6)	13.4
**female**	236,852 (48.4)	13.1
		
**Gestational age**		
**<37 weeks**	16,057 (3.3)	14.7
"term"	472,861 (96.7)	13.2
		
**Marital status of mother**		
**married**	376,222 (77.0)	10.1
**single**	112,696 (23.0)	23.9
		
**Time between births**		
**12 months or less**	19,987 (4.1)	16.0
**>12 months**	468,931 (95.9)	13.2
		
**Maternal Age**		
**17 years or less**	21,362 (4.4)	17.1
**18 to 34 years**	407,801 (83.4)	13.7
**35 years or more**	59,755 (12.2)	8.9
		
**Maternal Ethnicity**		
**caucasian**	351,051 (71.8)	14.3
**hispanic**	97,911 (20.0)	10.4
**black**	21,989 (4.5)	14.4
**other/missing**	17,967 (3.7)	8.5
		
**Maternal Educational level**		
**<high school**	85,284 (17.4)	23.9
**high school**	157,852 (32.3)	17.9
**some college**	111,824 (22.9)	10.5
**college**	112,384 (23.0)	3.0
**more than college**	13,840 (2.8)	1.7
**missing**	7734 (1.6)	12.5
		
**Maternal Alcohol use**		
**none**	477,733 (97.7)	12.6
**1 to 50 drinks/week**	11,185 (2.3)	42.8

We calculated adjusted odds ratios by analyzing smoking as a dichotomous exposure (mother smoked or did not smoke) and as a continuous variable (number of reported cigarettes per day during the pregnancy). Dichotomous outcomes of reported smoking during pregnancy yielded adjusted odds ratios of 1.9 (95% CI: 1.6 to 2.3) for death due to SIDS, 1.5 (95% CI: 1.3 to 1.7) for infant deaths from all causes, and 3.0 (95% CI: 1.4 to 6.3) for deaths due to respiratory etiologies (p < 0.01 for all outcomes). Table 2 shows results for the model for each of the outcomes. The final logistic regression model for each outcome was slightly different, but smoking remained in each as a significant factor. The interaction between ethnicity and cigarette use did not contribute significantly to the final model and thus was excluded.

**Table 2:** Adjusted odds ratios for SIDS, all infant deaths, and respiratory deaths with dichotomous cigarette use and other potential confounders*

	**SIDS**	**p**	**All deaths**	**p**	**Respiratory deaths**	**p**
**Reported smoking**		<0.0001		0.0001		0.0032
No cigarette use	1.0		1.0		1.0	
Cigarette use	1.9		1.5		3.0	
						

**Gender**		<0.0001		<0.0001	Not significant	
Female	1.0		1.0			
Male	2.0		1.4			
						

**Marital status**		<0.0001		<0.0001		0.0127
Married	1.0		1.0		1.0	
Not married	1.7		1.5		2.5	
						

**Pregnancy interval**		0.0033		0.0016		0.0056
12 months or more	1.0		1.0		1.0	
Less than 12 mo.	1.6		1.4		3.8	
						

**Gestational age**		0.0053		<0.0001	Not significant	
Term	1.0		1.0			
Less than 37 weeks	1.6		3.7			
						

**Any alcohol use**	Not significant		Not significant		Not significant	
						

**Education**		0.0002		<0.0001	Not significant	
More than college	1.0		1.0			
College	0.6		1.0			
Some college	0.9		1.2			
High school	0.9		1.2			
Some high school	1.3		1.4			
Unknown	0.7		2.1			
						

**Ethnicity**		0.0204	Not significant			0.0337
White	1.0				1.0	
Hispanic	0.8				1.8	
Black	1.4				0.6	
Other	0.7				4.5	
						

**Year of birth**		<0.0001		<0.0001	Not significant	
1998	1.0		1.0			
1997	1.7		1.0			
1996	1.7		0.8			
1995	1.6		0.8			
1994	1.7		0.9			
1993	2.7		1.0			
1992	3.0		1.1			
1991	3.6		1.3			
1990	3.0		1.3			
1989	3.1		1.4			

*Logistic regression modelling with corresponding p-values for each variable

Analyzing cigarette consumption as a continuous variable shows the associated odds *per cigarette *are 1.042 (95% CI: 1.030 to 1.054; p < 0.0001). In this model, consumption of 10 cigarettes per day is associated with a 51% increased odds of SIDS (95% risk limit 35% to 70%). The Hosmer-Lemeshow test statistic was non-significant (p > 0.1948) suggesting adequate model fit.

**Figure 1:**
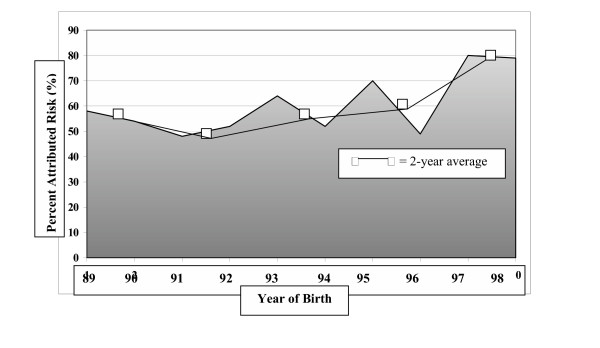
Percent attributed risk (PAR) between maternal prenatal smoking and sudden infant death, Colorado Birth Registry 1989–1998.

Table 3 shows crude rates of SIDS in the exposed and unexposed cohorts of infants for the years 1989 to 1998. Included is a calculated percent attributed risk (PAR) which, when multiplied by the number of SIDS deaths in the exposed cohort, yields the number of SIDS infants whose death is associated with maternal smoking ^12^. In this analysis, 101 infant deaths due to SIDS bear an association with maternal smoking among the exposed cohort of 172 infants. Figure 1 demonstrates the increasing PAR associating maternal prenatal smoking and SIDS during the study period, suggesting a stronger link between SIDS and maternal smoking. The average rate for each 2-year period is plotted in the Figure as well. Time was a highly significant variable in the study and SIDS rates decreased markedly over the study time period. The remaining SIDS deaths show a greater relative association with maternal smoking in the years following the *Back to Sleep *campaign. In the final year of the analysis, 80% of the SIDS deaths in the smoke-exposed infant cohort are attributed to maternal smoking.

**Table 3:** SIDS in smoke exposed and unexposed infants including attributed risk and deaths among smoke exposed infants by birth year, State of Colorado 1989–1998.

Birth year	SIDS deaths in:		Total SIDS	SIDS rate (SIDS/1000) in:		Relative Risk	PAR*	Deaths Attributed to smoking
	smoke exp	unexp		smoke exposed	unexp			
1989	21	51	72	3.21	1.33	2.4	58	12
1990	24	53	77	2.96	1.35	2.2	54	13
1991	27	69	96	3.32	1.72	1.9	48	13
1992	21	59	80	2.93	1.41	2.1	52	11
1993	22	49	71	3.28	1.17	2.8	64	14
1994	10	34	44	1.65	0.79	5.6	52	5
1995	12	28	40	2.19	0.65	3.4	70	8
1996	9	36	45	1.61	0.82	2.0	49	4
1997	16	29	45	3.21	0.64	5.0	80	13
1998	10	18	28	1.80	0.37	4.9	79	8

TOTAL:	172	426	598					101

* Percent Attributed Risk in smoke-exposed cohort

## Discussion

Smoking is deleterious and has negative effects on children born to mothers who smoke [12-16]. We confirm a two-fold increased risk of SIDS in infants born to smoking mothers. In addition, as one cause of SIDS (prone sleeping) was reduced through the *Back to Sleep *public health campaign in the United States, we noted an increasing attributed risk of SIDS with smoking. As a major etiology of SIDS, prone sleeping position, decreased over the study period due to the national educational campaign, the remaining SIDS deaths may now be due to the more isolated effects of tobacco exposure. Due to a decreased overall rate of SIDS, almost 80% of SIDS among smoke-exposed infants now bear a relationship to cigarette smoking.

Taylor and Sanderson, using data prior to the *Back to Sleep *campaign in the United States, suggest that up to 30% of SIDS deaths may be attributed to maternal smoking [17]. We note an increasing percent attributed risk in our cohort of 489,000 infants after the roll out of the campaign. Using the attributed risk to calculate an absolute mortality among the exposed cohort, 101 of 172 infant SIDS cases over the 10-year study period are linked to maternal smoking, which is greater than half of the infant deaths due to SIDS in the smoke-exposed cohort.

Our study cannot establish a causal role for maternal smoking in SIDS. Others have argued for a causal relationship based on prospective data, demonstrable dose-response relationships, and analysis using multiple potential confounders [18]. The causal path appears more likely on the basis of multiple studies that support such a link, consistent findings of a dose-response relationship in the literature, and a potential biological basis for the association between the exposure and the outcome [19]. In this study, removing one causative factor for SIDS, prone sleeping, may have increased the relative effect of another factor, maternal smoking, as an agent associated with SIDS.

The published literature reports a 20% to 30% smoking rate among pregnant women [20,21]. Approximately 20% of pregnant smokers will deny smoking, but when tested with urine cotinine will be positive [22]. A recent report suggests that 21% of women in Colorado are smokers [23]. In our cohort, 15% of women reported some smoking during pregnancy, which may indicate a degree of under-reporting consistent with the published literature. While about one-fourth of women quit smoking during pregnancy, the recidivism rate is high and women are most likely to continue smoking into the postnatal period [24-26]. Furthermore, as most individuals who smoke begin before the age of 18 years [27], the results reported here provide yet another clarion call to eliminate smoking initiation and remove this preventable risk factor for infant death and SIDS. Expectant mothers, women desiring pregnancy, and health care providers who care for them need to be reminded about this strong association of infant death with the preventable risk factor of maternal smoking. For example, physicians can counsel women desiring or planning pregnancy that if they smoke, the child will have a markedly increased risk of SIDS due to the smoking.

This study has several important limitations related to design and the nature of the data set used. Given the rich nature of the Colorado Birth Registry, we controlled for many potential confounding variables, but others may exist. This analysis likely represents an under-estimate of the actual associated risk with prenatal smoking, given that it relies on maternal report of a behavior widely known to be harmful. We suspect that self-report of maternal smoking underestimates the true rate of maternal smoking. Fetal and infant exposure to tobacco smoke occurs in manner ways, including prenatal maternal smoking, prenatal maternal second-hand smoke exposure, and postnatal smoke exposure by one or more care providers in the home. In this study we cannot distinguish among these exposure pathways, which may result in an erroneous under or over estimate of risk. The time period spanned by this study includes the rollout of the *Back to Sleep *campaign in the United States and our dataset did not assess whether parents positioned their infants prone or supine. We therefore could not control for this ecological association with SIDS. The very nature of retrospective data creates limitations in that we cannot carefully control the setting in which the data are collected, or the individual collecting the data. Missing data records are a third limitation, although this appears to have not been a major issue with this dataset.

## Conclusions

As prone infant sleeping has decreased, other causes of SIDS such as maternal smoking assume increased importance in the effort to protect infants from SIDS. Spanning the rollout of the *Back to Sleep *campaign in the United States we examined the increasing attributed risk associating smoking with SIDS. As SIDS rates declined, the attributed risk of SIDS with smoking has increased. In the final year of analysis, we demonstrated a link between 80% of the SIDS deaths and maternal smoking. A major, preventable exposure remains for infants in the United States and providers should redouble counseling efforts toward reducing this exposure.

## Competing interests

The author(s) declare that they have no competing interests.

## Authors' contributions

MA participated in the study conception, background research, study design, statistical analysis, and drafting of the manuscript. DJ participated in the study conception, study design, and proofreading of the manuscript. HB participated in the study conception, study design, statistical analysis, and proofreading of the manuscript.

## Pre-publication history

The pre-publication history for this paper can be accessed here:


